# Investigation of the physical, chemical and thermal properties of a novel lignocellulosic fiber extracted from the *Ravenala madagascariensis* leaf stalk

**DOI:** 10.1039/d5ra00644a

**Published:** 2025-07-16

**Authors:** Mohammad Abul Hasan Shibly, Mohammad Mohsin Ul Hoque, Prosenjit Sen, Khandaker Akil Mahadi Ohi, Md. Maruf Hossain, Md. Masum Mia, Md. Abdus Sabur, Mohammad Junaebur Rashid, Mohammad Mahbubur Rahman

**Affiliations:** a National Institute of Textile Engineering and Research Dhaka 1350 Bangladesh hasanduet002@gmail.com; b Jahangirnagar University Dhaka 1342 Bangladesh; c Bangladesh University of Textiles Dhaka-1208 Bangladesh; d Bangladesh Council of Scientific and Industrial Research Dhaka 1205 Bangladesh; e University of Dhaka Dhaka 1000 Bangladesh

## Abstract

Natural plant fibers are inexpensive, lightweight, renewable, and environmentally friendly, making them sustainable substitutes for synthetic materials. This study aims to identify alternative, eco-friendly replacements for nonbiodegradable fibers used in polymer composites. To achieve this goal, the fibers from *Ravenala madagascariensis* leaf stalks were thoroughly characterized, with a focus on their physical, mechanical, thermal, and morphological properties. The hygroscopic properties (moisture content and regain), density, and chemical composition of the fibers were evaluated following ASTM D2654, ASTM D1909, ASTM D891-18, and TAPPI standards, respectively. Chemical composition analysis revealed that the fiber contained 54.25 wt% cellulose, 20.12 wt% hemicellulose, and 15.17 wt% lignin, contributing to its enhanced mechanical properties. The crystallinity, surface structure, chemical bonds, and thermal behavior of the fibers were analyzed *via* XRD, SEM, FTIR, and TGA techniques. This novel fiber has a moisture content and regain percentages of 9.17% and 10.1%, respectively. Its average tensile strength is 151 MPa for a 20 mm gauge length (GL) and 136.8 MPa for a 30 mm gauge length (GL), with a crystallinity index of 67.37%, in which the size of the crystals is 15.64 nm. The fiber degradation begins at a maximum temperature of 550 °C. This original fiber holds potential for applications in the production of cellulose nanoparticles, fiber-reinforced composites, biomaterials and so on.

## Abstract 2

Natural plant fibers are inexpensive, lightweight, renewable, and environmentally friendly, making them sustainable substitutes for synthetic materials.

## Graphical abstract

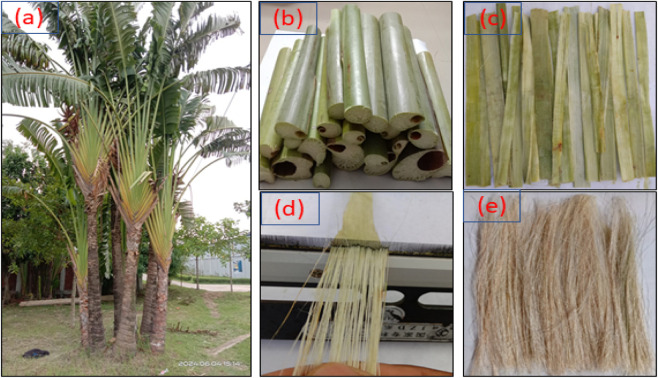

## 1. Introduction

Over the past three decades, the development of fiber materials has flourished substantially because of their wide variety of applications. For instance, the utilization of synthetic fibers in composite products is remarkable due to their useful contributions to supplementary products as well as their high-strength material qualities. However, the high carbon emissions while producing such fibers as well as the awareness of ‘zero carbon emissions’ in recent years have shifted the focus to produce eco-friendly composite materials.

Environmental threats, along with protective regulations, have served as catalysts for the use of natural resources across various production sectors.^1,2^ As a result, interest in finding new materials to replace traditional materials is increasing, with natural fibers emerging as promising options. Note that natural fibers are biodegradable, recyclable, and lightweight. Moreover, its natural polymers do have appealing fiber properties, such as a wide stiffness range and a high strength-to-weight ratio.^3^ Owing to their natural abundance, exploring more opportunities to find sources of natural fibers is essential.^4^

Recent studies suggest that natural fibers are well suited for reinforcing polymeric composite materials; thus, natural fibers are replacing synthetic fibers in the composite industry.^5–12^ The utilization of natural fibers in industry and agriculture, however, results in the production of significant amounts of waste.^13–15^ Therefore, developing an efficient method to convert biomass waste into usable reinforcement materials may provide a solution for producing economical and environmentally friendly composites. The “green composites”^16,17^ are made from natural fiber reinforcements, and several researchers have already produced such composites.^18,19^ For example, banana fibers^20^ are extensively used in textiles to provide a lustrous appearance. Furthermore, nontraditional fibers such as hemp and flax^21,22^ have become popular and are gradually replacing synthetic fibers.^23^

In fact, natural fibers consist of cellulose, hemicellulose, lignin, and pectin, with the proportions of these components varying between different fibers.^24^ A higher cellulose content increases fiber flammability, whereas a greater lignin content tends to lower the fiber degradation temperature.^21,22,25^ As a result, the presence of lignin and cellulose enhances the thermal stability of natural fibers and their functionalized materials, making them suitable for use in various polymer matrices for diverse functional applications.^23,26^ Additionally, natural fibers offer an alternative energy source for biodegradable reinforcement materials.^27^ The strong chemical and electrical resistance, effective thermal and acoustic insulation, and high fracture resistance of these materials make them an attractive area of research for various potential applications.^28^ Nearly every industry is moving toward a greener, eco-friendly approach, aiming to replace synthetic materials with natural alternatives.^29^ Many automotive manufacturers now use biofiber-based composites, such as headliners, trunk liners, dashboards, seat backs, and door panels, to produce various car parts and accessories.^30,31^ Additionally, fiber-reinforced composites are also used in the shipbuilding, aerospace, and construction industries.^32^Table 1 lists the applications and sources of different plant-based natural fibers.

**Table 1:** Different plant fibers and their applications

Fiber name	Source	Scientific name	Applications	Reference
Hemp	Hemp plant	*Cannabis sativa*	Textiles, paper, ropes, and skincare products	33–36
Ramie	Ramie plant	*Boehmeria nivea*	Apparel, home furnishings, and fishing nets	37, 38
Pineapple leaf fiber	Pineapple leaves	*Ananas comosus*	Textiles, upholstery, accessories	39, 40
Typha fiber	Typha leaves	*Typha Australis*	Composites	41
Hogla	Hogla plant	*Typha elephantina Roxb*	Textiles, and composites	42
Coconut tree	Primary flower leaf stalks, husks	*Cocos nucifera*	Composites, rope, doormats, gardening products	43, 44
Palm tree	Leaf stalks	*Livistona rotundifolia*	Composites, packaging materials	45
Corn leaf fiber	Corn plant	*Zea mays*	Composites	46, 47
Kenaf	Kenaf plant leaf stalks	*Hibiscus cannabinus*	Packaging materials, insulation	48, 49
Stinging nettle fiber	Stinging nettle plant	*Urtica dioica*	Clothing, cordage, twine	50, 51
Water hyacinth fiber	Water hyacinth plant	*Pontederia crassipes*	Polymer composites	52–54
Banana fiber	Banana plant stems	*Musa* spp.	Textiles, handicrafts, papermaking	55–57
Soy silk	Soybean residue	*Glycine max*	Clothing, accessories	58
Piñatex	Pineapple leaves	*Ananas comosus*	Textiles, footwear, bags, accessories	59
Sisal	Sisal plant leaves	*Agave sisalana*	Ropes, twine, carpets, geotextiles	60
Abaca	Abaca plant (Manila hemp)	*Musa textilis*	Fiber craft, tea bags, specialty papers	61
Lotus fiber	Lotus plant rhizomes	*Nelumbo nucifera*	Luxury textiles, traditional asian garments	62
Kapok	Kapok tree seeds	*Ceiba pentandra*	Pillow stuffing, insulation, and hydrogel	63
Spider silk	Produced by spiders	*Various spider species*	Lightweight but strong textiles, medical uses	64, 65

A wide variety of natural fibers are derived from various parts of plants, including the stem, root, bark, leaf stalk, husk, fruit, *etc.*^66^ The literature review highlighted the importance of characterizing new natural fibers for composite materials, as such analysis determines their potential for various applications. During this process, a previously uncharacterized cellulosic natural fiber was identified in the leaf stalk of the *Ravenala (R.) madagascariensis* plant. Since no prior studies on the characterization of this specific fiber were found in the existing literature, the present study conducted its characterization based on established methodologies used for other natural fibers The main objective of this study was to examine the physical, chemical, thermal, and mechanical properties of *Ravenala madagascariensis* fibers (RMFs). The investigations included the linear density, moisture properties, chemical composition, surface morphology, functional characteristics, crystallinity index, crystallite size, mechanical properties, and thermal behavior of the RMFs. These properties were analyzed *via* chemical analysis, scanning electron microscopy (SEM), Fourier transform-infrared (FT-IR) spectroscopy, X-ray diffraction (XRD), tensile testing, and thermogravimetric analysis (TGA).

## 2. Materials and methods

### 2.1 Materials

*R. madagascariensis* is a tree that can grow at a height of 30–60 feet (9–18 m). Its leaves are green, oblong, and feature a broad, pinnate margin with evergreen venation, measuring over 2.4 meters in length. Globally, *R. madagascariensis* is native to Madagascar, where it grows both in the wild and in cultivated settings. It is also widely cultivated in tropical and subtropical regions—including Bangladesh, India, and parts of North America—mainly for ornamental landscaping due to its striking appearance.^67^ In this study, lignocellulosic fibers were extracted from the leaf stalk of the *R. madagascariensis* plant through an eco-friendly extraction process. Currently, there is no comprehensive data on the global annual harvest of Ravenala madagascariensis for fiber use. While native to Madagascar and occasionally traded for ornamental purposes, it is not commercially harvested at scale for its fiber. Notably, 90 kg of its seeds were exported to Pakistan in July 2021, indicating limited international trade. This study focuses on the basic fiber characterization of the plant, aiming to support future research into its commercial viability and sustainable fiber applications.^68^

### 2.2 Extraction of the fibers

*Ravenala madagascariensis* is an abundant ornamental plant widely cultivated in tropical and subtropical regions, including Bangladesh. Its large leaf stalks are regularly pruned to maintain the aesthetic appearance of the plant, resulting in a substantial amount of biomass waste. Instead of discarding these stalks, they were collected and chopped into 10′′–12′′ pieces and prepared for fiber extraction. This approach offers a sustainable solution for waste valorization while contributing to the development of environmentally friendly, renewable materials for composite applications. This study presents the first detailed characterization of fibers derived from this plant species, revealing promising attributes for utilization in textile and biocomposite applications.

**Fig. 1:**
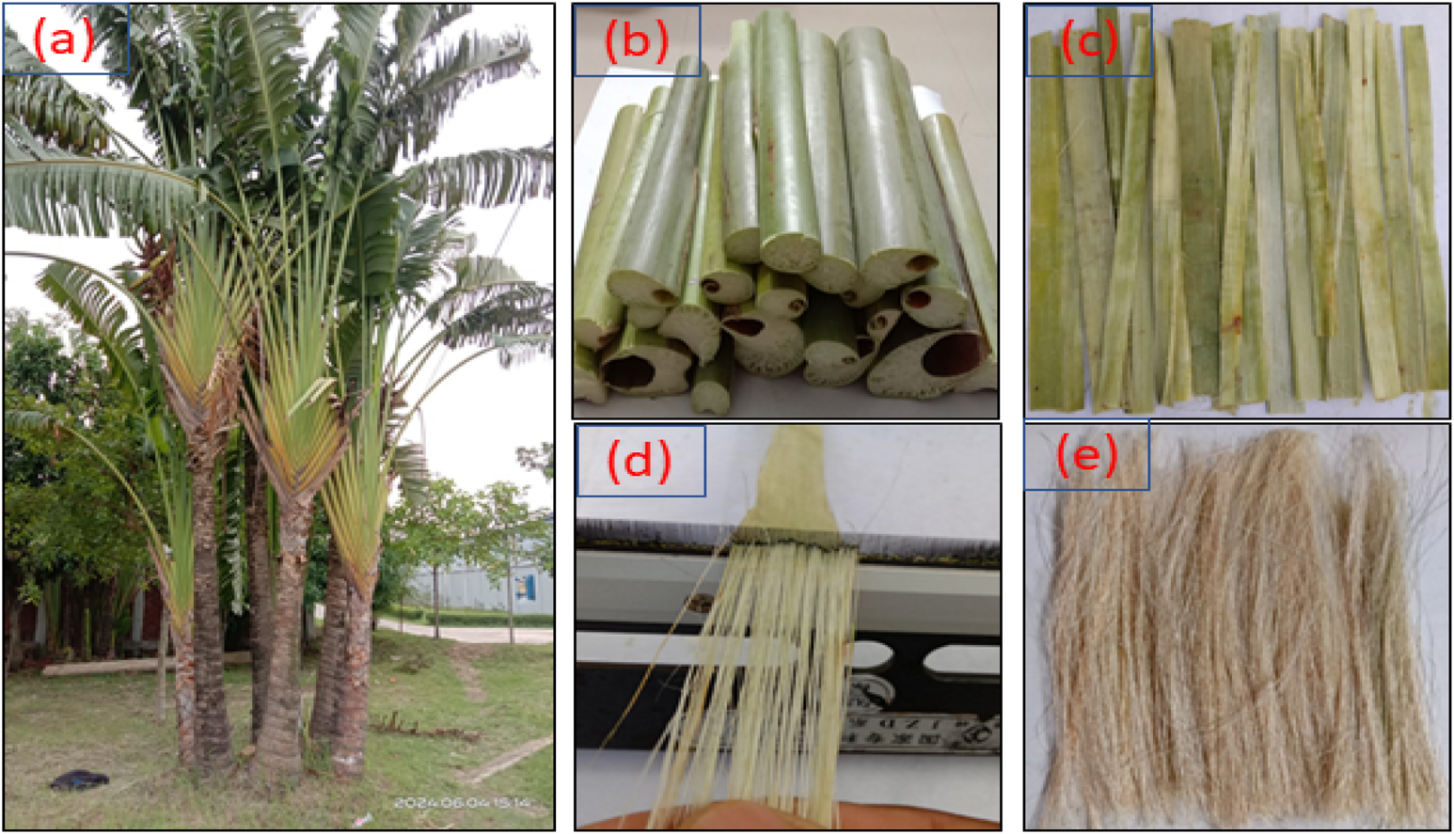
RMF extraction process: (a) *R. madagascariensis* plant, (b) collected leaf stalks, (c) removed spongy parts of leaf stalks, (d) mechanical retting, and (e) extracted fibers.

A variety of natural fibers are obtained from different parts of plants through methods such as water retting, mechanical processes, chemical extraction and enzymatic retting. The selection of an extraction method is influenced by factors such as the type of fiber needed, the method's efficiency, the time available, the intended application of the fibers, and the cost of extraction.^69^ The leaf stalks of *R. madagascariensis* were cut, and the hollow sections inside them were removed. The fibers were then extracted from the leaf stalks *via* a mechanical combing process, as shown in Fig. 1. The extracted fibers were washed at a temperature of 50 °C for 2 hours to remove excess gummy substances, and their surfaces were thoroughly cleaned with fresh water. After washing, the fibers were dried under direct sunlight for approximately two days.

### 2.3 Methods of fiber characterization

#### 2.3.1 Physical characterization and fiber density measurement

The *R. madagascariensis* fibers were conditioned under standard conditions (20 °C temperature and 65% relative humidity) for 48 hours. Fiber diameters were measured at three random points on 22 fibers *via* an optical microscope, and longitudinal images were captured from different fiber samples. The average diameter was calculated using the “Image-Pro Plus” software.^70^ The linear density of the fibers was determined in Tex units following ASTM D 1577-92, with measurements taken from 25 individual fibers to calculate the average count. The fiber density was measured *via* a pycnometer setup with toluene, a liquid of known density, and the fiber density was calculated *via* the following equation:^71^1
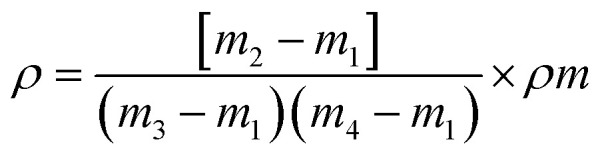
where *ρ* is the density of the RMF (g cm^−3^) and *ρm* is the density of methanol (g cm^−3^). m1: the mass of the empty pycnometer (g). m2: mass of the pycnometer filled with methanol (g). m3: mass of the pycnometer filled with chopped RMFs (g). m4: mass of the pycnometer filled with chopped RMFs and methanol (g)

#### 2.3.2 FTIR analysis

Fourier transform infrared (FTIR) spectroscopy (FT-IR Spectrum II, PerkinElmer, Llantrisant, UK) was used to analyze the active chemical components of the *R. madagascariensis* fibers. The fibers were ground into a fine powder and mixed with transparent potassium bromide (KBr) for infrared measurement. The FTIR spectrometer was operated in absorbance mode at a room temperature of 30 °C and a relative humidity of 65%, with a scan rate of 32 per min, and the resolution was 2% within the wavenumber range of 500–4000 cm^−1^.

#### 2.3.3 Moisture content and regain

The moisture content (MC) and moisture regain (MR) percentage were determined using the ASTM D 2654 and ASTM D 1909 methods, respectively. A 5 g fiber sample was tested under standard atmospheric conditions of 20 °C and 65% relative humidity. The weighed samples were placed in an air oven maintained at a constant temperature of 105 °C. The sample weight was recorded at 15-minute intervals until the change in weight between successive measurements was less than 0.1%. The difference between the standard conditioned weight and the oven-dry weight was used to calculate the moisture content and moisture regain of the RMFs. The calculations were performed *via* the following equation.2
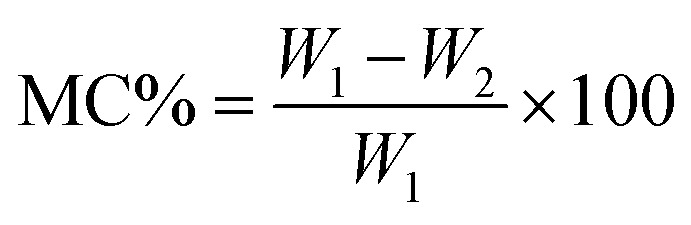
3
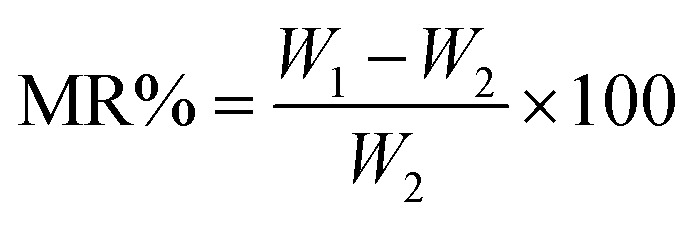
where *W*_1_ and *W*_2_ denote the fiber weights before and after drying, respectively, in grams.

#### 2.3.4 Mechanical property analysis

The tensile strength, Young's modulus and elongation at break of the fibers were measured following ASTM D 3822-07 standards. Tests were conducted at room temperature using a Hounsfield H10KS testing machine (UK) with a crosshead speed of 10 mm min^−1^ and two fiber GLs of 20 mm and 30 mm under a relative humidity of 65 ± 3%. For each GL, 11 RMFs were tested, and the average results were recorded. A 1.0 kN load cell was used to measure the force applied during the tests.^72^ The fiber diameter and tensile strength were statistically analyzed *via* the Weibull distribution with Minitab statistical software 2022. The tensile strength and Young's modulus were calculated *via* the following equation:4
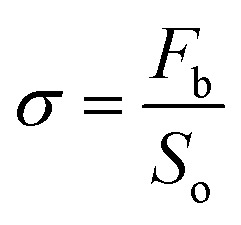
where *F*_b_ and *S*_o_ denote the maximum force at break and the cross-sectional area of the fibers, respectively.

#### 2.3.5 XRD analysis

The crystallinity index of the RMF sample was analyzed using X-ray diffraction (XRD). This index, which reflects the degree of structural organization, is crucial because it affects the alkali treatment process and the mechanical properties of natural cellulose fibers. The analysis was performed with a BRUKER AXS Diffractometer D8 (Germany) utilizing Cu Kα radiation under operating conditions of 40 kV and 40 mA. The diffracted X-rays were scanned using the detector provided in the diffractometer, covering an angular range of 5° to 60° (2*θ*) at a scan speed of 3°min^−1^ with 0.02° step increments. The crystallinity index (CI) of the RMF was calculated using empirical methods, as described by the following equation:5
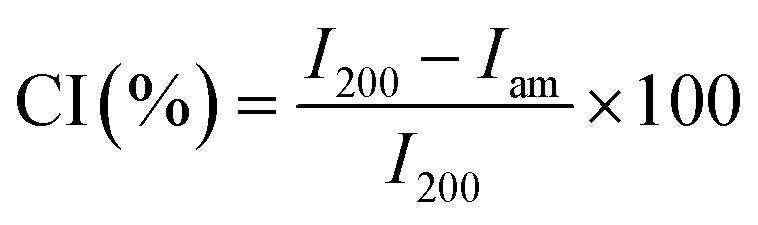
In this context, *I*_200_ represents the maximum intensity of the peak at a 2*θ* angle between 22° and 23°, corresponding to the crystalline region. Similarly, *I*_am_ denotes the minimum intensity of the peak at a 2*θ* angle between 15° and 19°, which represents the amorphous region. The crystallite size of the RMFs was determined *via* Scherrer's equation, as outlined below:6
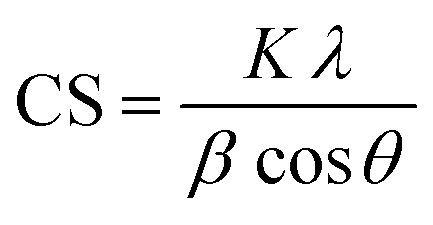
*K* represents Scherrer's constant with a value of 0.89, whereas *λ*, *θ* and *β* correspond to the wavelength of the radiation, the Bragg angle, and the full width at half maximum (FWHM), respectively.

#### 2.3.6 TGA along with DSC analysis

Thermogravimetric analysis (TGA) was conducted to assess the thermal stability of the fibers using an SDT650 thermal analyzer, which integrates TGA and DSC functions, from TA Instruments, USA. TGA is critical for evaluating the thermal durability of natural fiber components and determining the operational temperature range for composites incorporating such fibers. For this analysis, 10 mg of RMF was used. The TGA and DSC experiments were carried out in a nitrogen atmosphere, with the temperature increasing from room temperature to 600 °C at a constant heating rate of 10 °C min^−1^ and a nitrogen flow rate of 30 ml min^−1^.^73^ The kinetic activation energy (*E*_a_), which represents the minimum energy required to degrade the fiber, was determined using Broido's equation.

Differential scanning calorimetry (DSC) was performed to complement the thermogravimetric analysis. A 10 mg sample was placed in sealed pans to prevent contamination. The sealed pan was then positioned inside the calorimeter and heated under an inert nitrogen atmosphere up to 45 °C. Significant melting peak temperatures were recorded at a consistent heating rate of 10 °C min^−1^.^74^

#### 2.3.7 SEM and EDX analysis

The surface and cross-sectional morphologies of the RMFs were examined through scanning electron microscopy (SEM) using a JEOL 6460LV instrument (Tokyo, Japan). The analysis was conducted at an accelerating voltage of 20.0 kV. Prior to testing, the samples were coated with gold under vacuum to increase their conductivity.

Energy dispersive X-ray spectroscopy (EDX) is a widely used method for identifying surface elements, such as oxygen, nitrogen, and carbon, in natural fibers. EDX analysis, which was conducted *via* the TEAM™ EDS system integrated with SEM, was employed to identify the elemental composition of the *R. madagascariensis* fibers.

#### 2.3.8 Chemical composition analysis

The chemical composition of RMFs was analyzed *via* TAPPI standard methods. The lignin content was determined according to TAPPI T211 om-8324. The extractive content of the fibers was determined following the TAPPI T204 om-88 standard method. Holocellulose and cellulose contents were measured following the TAPPI T249 and TAPPI T203 om-93 methods, respectively.^75^ The hemicellulose content was calculated *via* the following equation:

7 Hemicellulose% = Holocellulose% − Cellulose%

## 3. Results and discussion

### 3.1 Physical characterization and fiber density

**Fig. 2:**
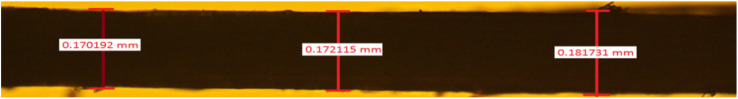
Optical microscope image of the RMF.

The *R. madagascariensis* leaf stalk fibers had an average length of 26.4 cm. Accurately measuring the diameter of natural fibers is challenging because of their irregular thickness, which varies along their length as a result of environmental factors and growth conditions. To determine the diameter of *R. madagascariensis* fibers, measurements were taken at three random points on each fiber. As shown in Fig. 2, the diameters at the first, second, and third points were 0.170192 mm, 0.172115 mm, and 0.181731 mm, respectively. The average single-fiber weight was calculated as 0.008455 g on the basis of 20 fibers of varying lengths. The fineness of the fiber was 33.70 ± 11.72 Tex. Table 2 shows the fineness of *Rosa hybrida* bark fiber (14.51 Tex), *Hylocereus undatus* stem fiber (14.82 Tex), and *Saccharum bengalense* grass fiber (18.63 Tex) which are all lower than RMFs. The specific density was 1.08 g cm^−3^. This low density makes it suitable for lightweight applications as an alternative to synthetic fibers. A comparison of the physical properties of RMFs with those of other natural fibers is shown in Table 2.

**Table 2:** Comparison of the physical properties of RMFs with different natural fibers

Fiber	Diameter (μm)	Density (gm per cc)	Count (Tex)	Reference
RMFs	140–303	1.08	33.70 ± 11.72	Current work
*Rosa hybrida* bark fiber	214–238	1.194	14.51	66
Coconut tree leaf	140–990	1.2	—	72
*Hylocereus undatus* stem fiber	173.53	1.08	14.82	76
*Eleusine indica* grass	315.4 ± 10	1.14	—	77
*Cyperus pangorei* fiber	133.3	1.10	11–14	78
*Saccharum Bengalense* grass	320.47	1.17	18.63 ± 6.28	79
*Ziziphus mauritiana* fiber	570.2	1.13	—	80

### 3.2 FTIR analysis

**Fig. 3:**
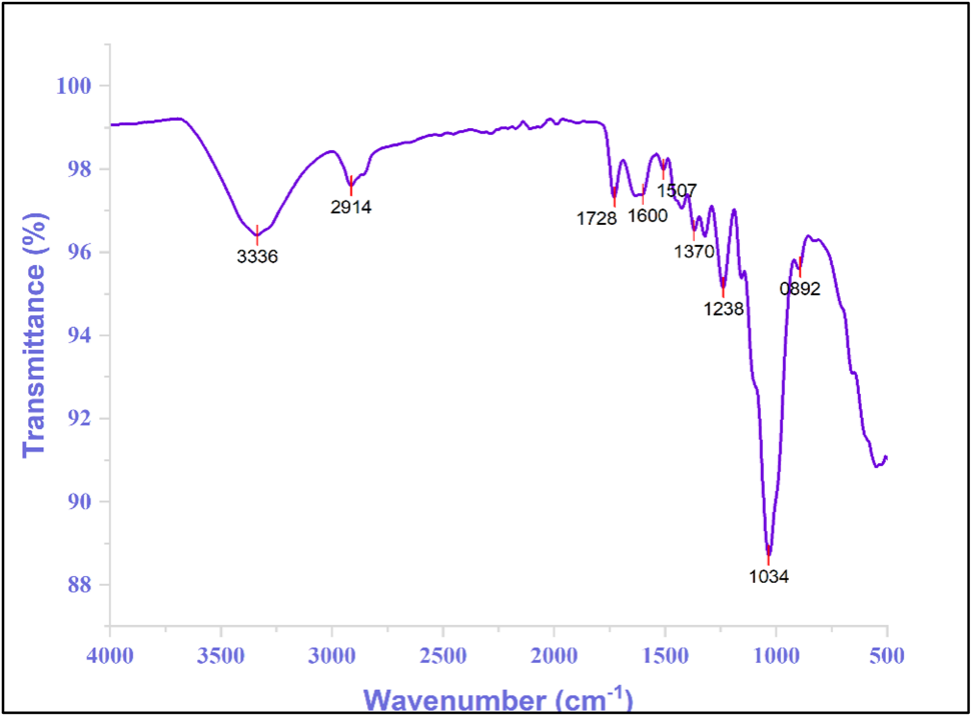
FTIR spectrum of *R. madagascariensis* fiber.

The Fourier transform infrared (FTIR) spectrum shown in Fig. 3 presents the transmittance (%) as a function of wavenumber (cm^−1^). The spectrum exhibited several significant absorption bands corresponding to specific molecular vibrations. Key peaks are observed at 3336 cm^−1^, 2914 cm^−1^, 1728 cm^−1^, 1600 cm^−1^, 1507 cm^−1^, 1370 cm^−1^, 1238 cm^−1^, 1034 cm^−1^, and 892 cm^−1^. The prominent peak at approximately 3390 cm^−1^ is typically associated with O–H stretching vibrations, indicating the presence of hydroxyl groups or water molecules. The peak regions of the fibers corresponding to the specified functional groups are presented in Table 3.

**Table 3:** Chemical stretching patterns associated with peak positions

Wavenumber (cm^−1^)	Allocations	References
3336	A prominent absorption peak at 3336 cm^−1^ is observed in RMFs, attributed to the O–H and C–H stretching in cellulose. This peak corresponds to the presence of alpha-cellulose, polysaccharide, and monosaccharide molecules	81
2914	The small peak at 2914 cm^−1^ is associated with the C–H stretching vibrations of CH and CH_2_ groups in cellulose and hemicellulose	82
1728	The small peak observed at 1728 cm^−1^ is attributed to the carbonyl group of carboxylic acid present in lignin	83
1600	A small peak in 1600 cm^−1^ is indicating water absorption in natural cellulose	84
1507	The vibrational activity at 1507 cm^−1^ is attributed to the stretching of C=C bonds in aromatic lignin structures	85
1370	The C–O groups in the aromatic rings of hemicellulose and lignin	86
1238	The absorbance peak at 1238 cm^−1^ corresponds to the C–O stretching vibration of an acetyl group in lignin	87
1034	The absorption peak at 1034 cm^−1^ is attributed to the C–O vibration in cellulose	88
892	Hemicellulose exhibits characteristic peaks at 896 cm^−1^, corresponding to the stretching vibrations and deformations of C–C–H, C–O–C, and C–C–O bonds in cellulose	89

### 3.3 Moisture content and regain analysis

The composition of a fiber is significantly affected by its moisture content. The ability of a textile to retain body heat under varying climatic conditions greatly influences its comfort level, making moisture regulation a critical aspect of performance. Changes in moisture content impact textile properties such as elasticity, friction, fiber diameter, and tensile strength. A decrease in equilibrium relative humidity can cause a textile to become weaker, more brittle, and fragile. To minimize moisture loss to the environment, maintaining air humidity during fiber processing is essential.^90^ The average moisture content and moisture regain of the fibers are 9.172% and 10.102%, respectively, which are similar to those of bamboo fibers (9.16%).^91^ The results were obtained *via* the use of five distinct fiber samples, each weighing five grams. The standard deviations for the two measurements were 0.601 and 0.726, with coefficients of variation of 6.56% and 7.18%, respectively, indicating consistent moisture contents and regain values among the samples.

### 3.4 Fiber mechanical property analysis

**Fig. 4:**
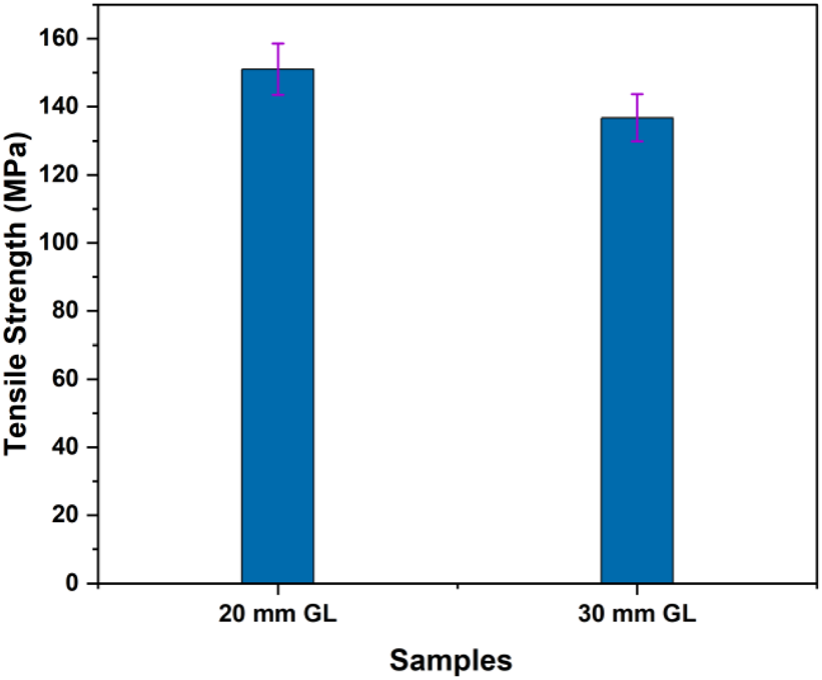
Tensile strength of *R. madagascariensis* fiber.

The mechanical properties of the RMFs were measured through tensile tests conducted for two different gauge lengths (GLs), as detailed in Table 4. Three key tensile properties were analyzed: tensile strength, Young's modulus and elongation at break. As illustrated in Fig. 4, the tensile strength of the 20 mm GL was significantly greater than that of the 30 mm GL. Factors such as the fiber extraction method, leaf age, climate conditions, microstructure, and defects caused by cracks influence the tensile properties of RMFs. The presence and accumulation of defects in the longer GL (30 mm) led to more rapid failure. Additionally, the tensile test results are impacted by the GL, instrument precision, grips, and compliance of the testing device.^66^

**Table 4:** Summary of the mechanical properties of *R. madagascariensis* fibers

GL (mm)	Mean diameter (mm)	Tensile strength (MPa)	Young's modulus (GPa)	Elongation at break (%)
20	0.2191 ± 0.05	151 ± 19.99	4.325 ± 1.36	6.475 ± 3.63
30	0.1933 ± 0.05	136.8 ± 26.64	5.70 ± 1.15	5.178 ± 2.89

**Fig. 5:**
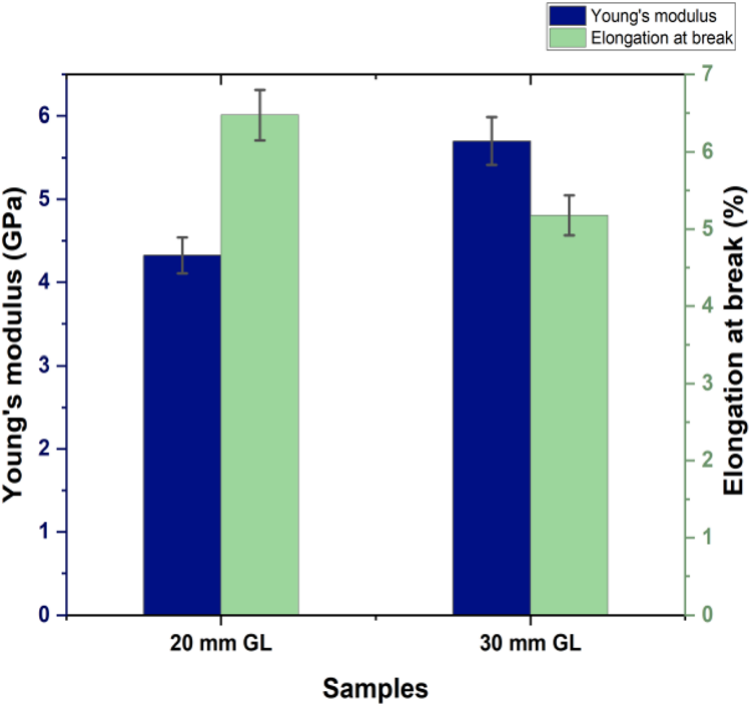
Young's modulus and elongation at break of *R. madagascariensis* fiber.

**Fig. 6:**
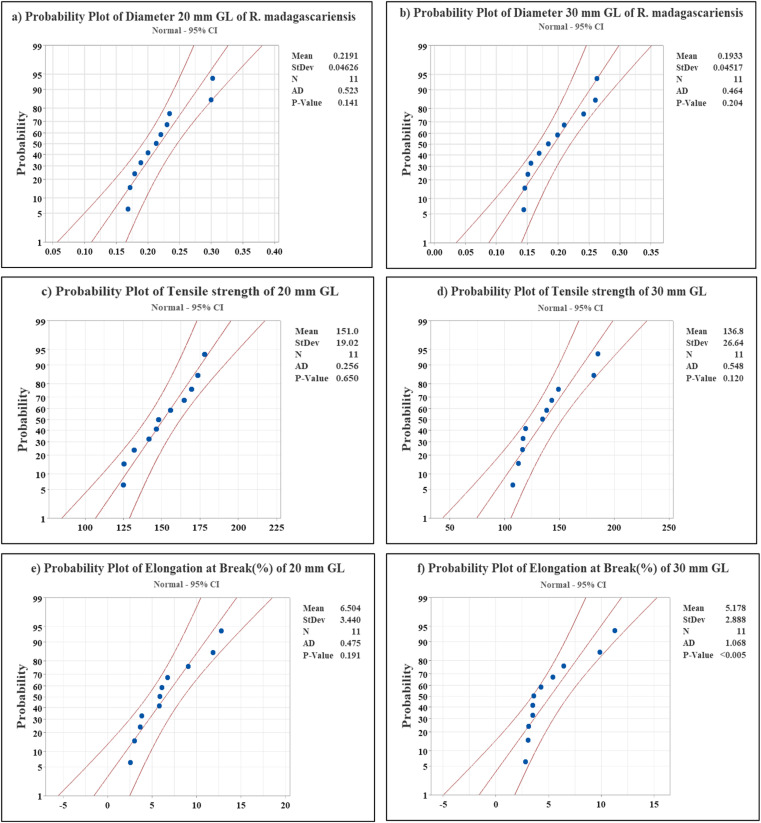
Weibull distribution plot; (a) diameter (20 mm GL), (b) diameter (30 mm GL), (c) tensile strength (20 mm GL), (d) tensile strength (30 mm GL), (e) elongation at break (20 mm GL), (f) elongation at break (30 mm GL).

The results in Fig. 5 demonstrate that the Young's modulus for the 30 mm GL is 5.70 GPa greater than that for the 20 mm GL. This trend is expected, as the arrangement of defects relative to the fiber length and volume may result in an increase in the Young's modulus with increasing GL.^66^Fig. 6 presents the Weibull distribution plots for the diameter, tensile strength, and elongation at break of the RMFs. The data show that the values for diameter, tensile strength, and elongation at break fall within the expected range and align well with the fitted curves. This study concludes that the mechanical properties determined using the two-parameter Weibull distributions closely match the average experimental results.

### 3.5 XRD analysis

**Fig. 7:**
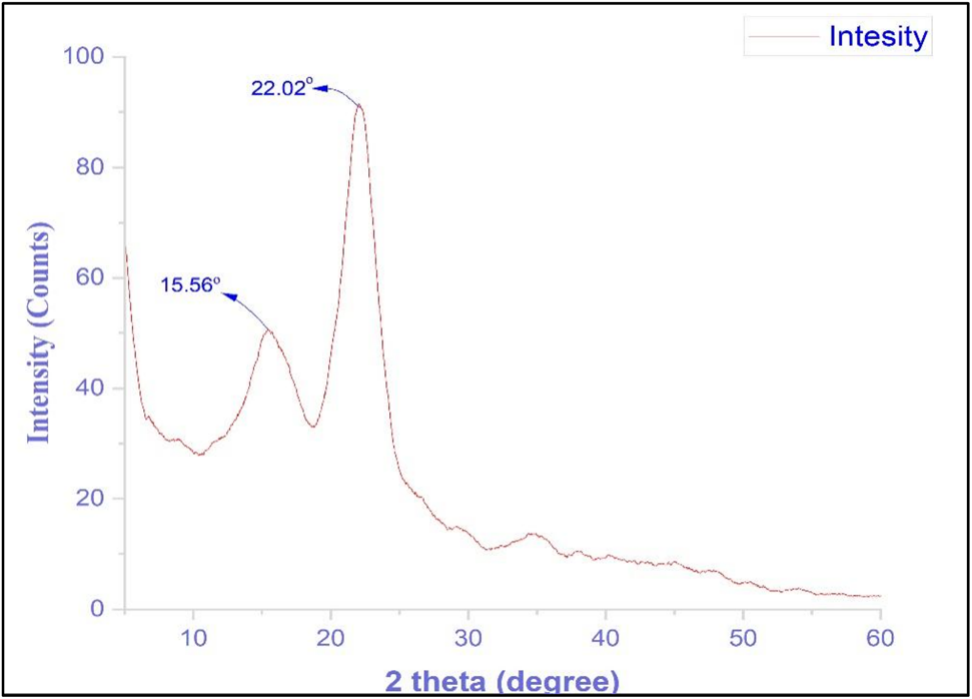
X-ray spectra of *R. madagascariensis* fiber.

Natural cellulose exists in two forms, Iα and Iβ. Certain plant fibers, such as cotton, jute, flax, and hemp, tend to have a relatively high proportion of Iβ. Beta cellulose is formed by eliminating water and forming oxygen bridges between C-1 and C-4, with the stacking of parallel hydrogen-bonded sheets partially stabilized by van der Waals interactions.^92^ The X-ray diffraction (XRD) pattern of natural fibers extracted from *R. madagascariensis* is shown in Fig. 7. The *x*-axis, labeled “2 theta (degree),” ranges from 5 to 60°, whereas the *y*-axis represents “intensity (counts).” The pattern reveals crystalline regions with peaks at specific 2*θ* angles. Two prominent peaks are observed: a broad and intense peak at 2*θ* = 15.56° and a sharp, tall peak at 2*θ* = 22.02°, indicative of a strong crystalline phase corresponding to cellulose-I diffraction.

The crystallinity index (CI) of the *R. madagascariensis* fibers was 67.37%, which was higher than that of the *Dracaena reflexa* fibers (57.32%), Coir fibers (57%), *Calotropis gigantea* fibers (56.08%), *Grewia tilifolia* fibers (41.7%) and Palmyra seed sprout fibers (PSSFs) (38%). Fibers with more crystalline regions exhibit enhanced mechanical stability and stiffness. However, the moderate CI of this fiber suggests that its mechanical strength and stiffness are also moderate. The CI implies that the fiber is thermally stable and can withstand a broad temperature range before degradation. The amorphous fraction (32.63%) makes the fibers more susceptible to swelling and disintegration in certain solvents. The higher crystallinity index indicates that the fiber may be suitable for biocomposite materials requiring a specific amorphous-to-crystalline ratio.^93,94^

The average size of a single crystal, referred to as the crystalline size (CS), was calculated to be 15.64 nm. In materials science, the crystalline size is commonly used to describe nanoparticles, colloids, gels, and spray-dried agglomerates. A smaller crystallite size enhances the sintering process, allowing for lower sintering temperatures.^95^

### 3.6 TGA along with DSC analysis

**Fig. 8:**
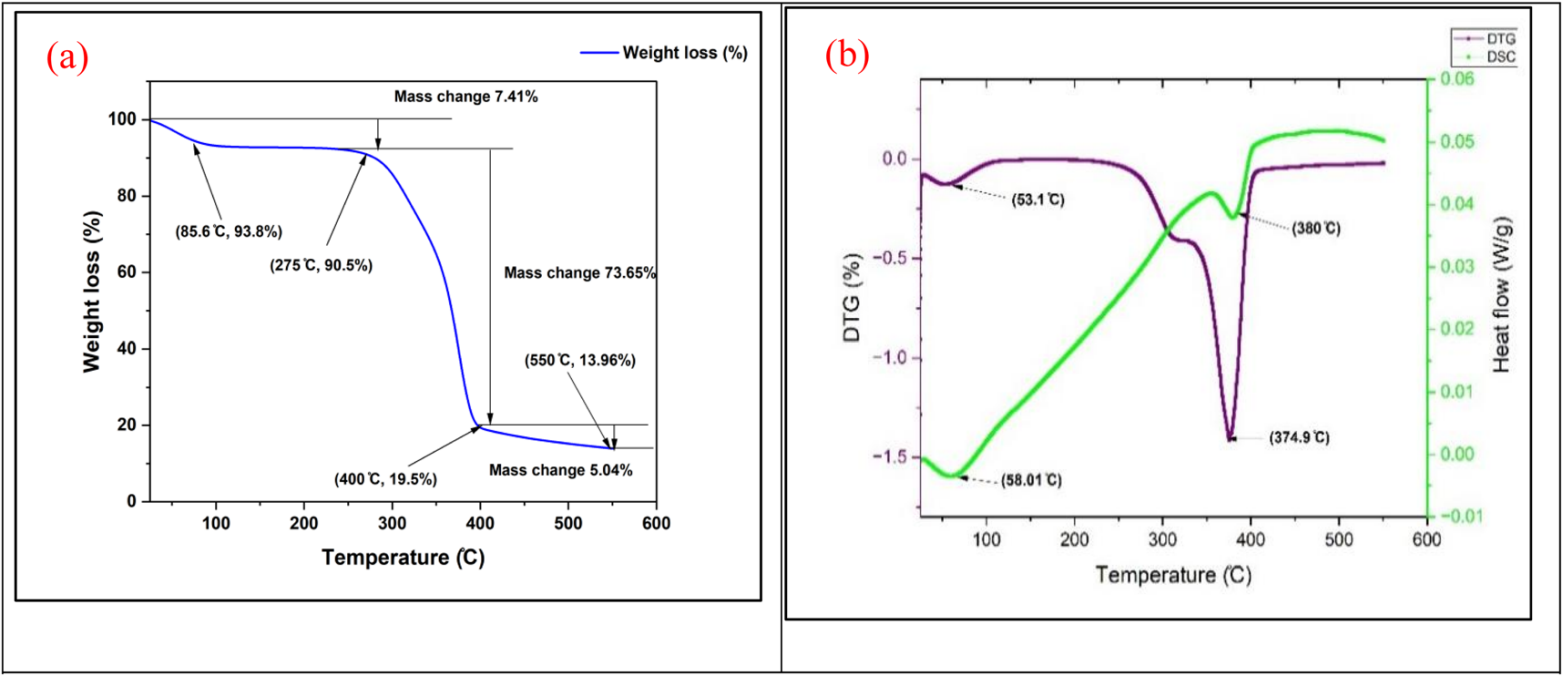
TGA (a), DTG and DSC (b) study of *R. madagascariensis* fiber.

Fig. 8 shows the thermogravimetric behavior of the RMF sample, along with the corresponding differential scanning calorimetry (DSC) data. The graph depicts the mass change of the sample as a function of temperature or time. The percentage weight reduction, relative to the sample's initial weight, is plotted on the right *y*-axis, whereas the heat flow in watts per gram (W g^−1^) is shown on the left *y*-axis. The *x*-axis represents the temperature in degrees Celsius (°C). The data points on the graph are marked with brackets and connected by lines. As the temperature increased, the sample weight decreased, indicating disintegration. A more complex heat flow curve is also observed.

The weight reduction begins significantly at 85.6 °C, reaching 93.8%, likely due to the evaporation of moisture or volatiles. Dehydration is suggested by the first endothermic peak at 58.01 °C, with a heat flow of −0.003 W g^−1^. Effective decomposition occurs at 275 °C, with a weight loss of 90.5%. The most prominent thermal event is an exothermic peak at 380 °C (0.037 W g^−1^), indicating further degradation. At 400 °C, a substantial weight reduction of 19.5% was observed, indicating the onset of decomposition. Finally, the material continues to degrade or transform at 550 °C, as shown by a weight loss of 13.96% and a heat flow of 0.05 W g^−1^.

### 3.7 Morphological analysis (SEM and EDX)

**Fig. 9:**
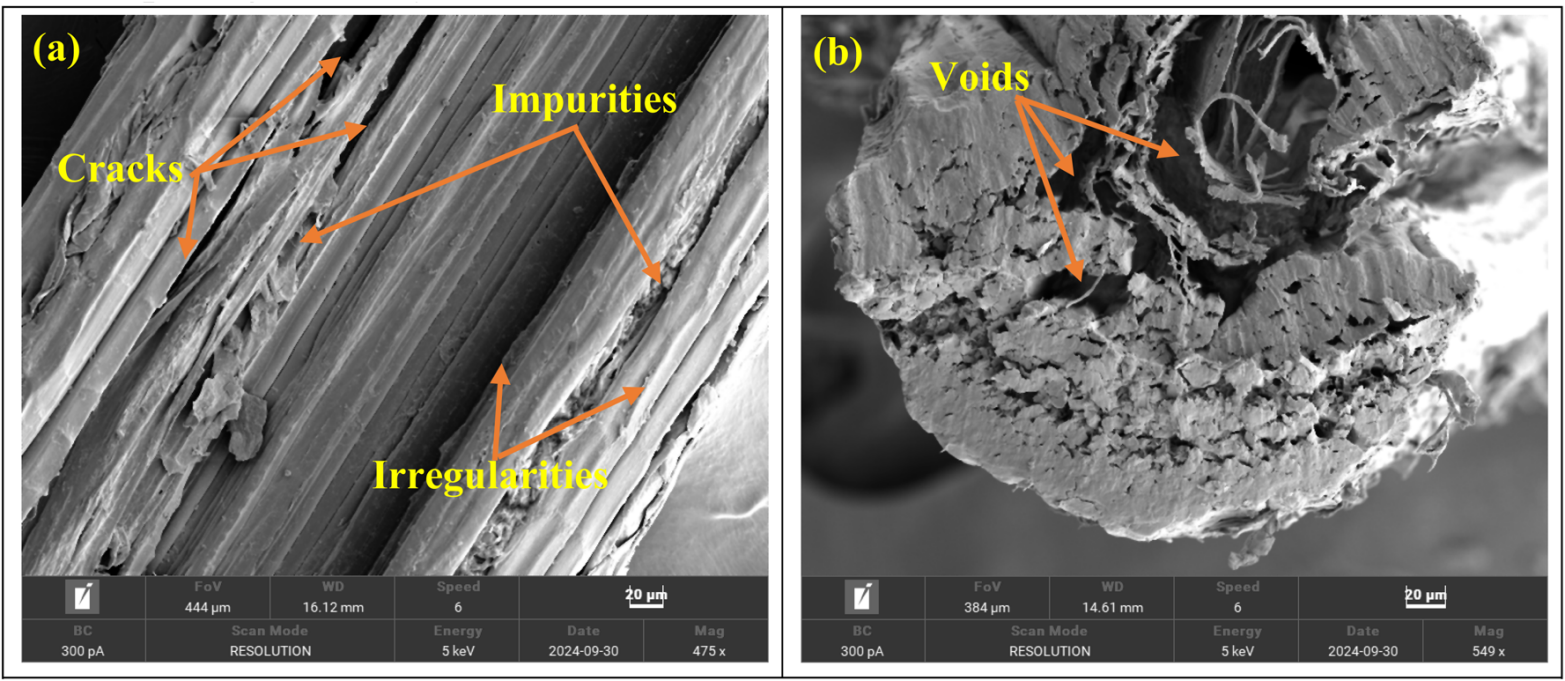
SEM images of the longitudinal views (a) and cross-sectional views (b) of RMFs.

SEM was used to examine the surface morphology of the untreated *R. madagascariensis* fibers. As represented in Fig. 9a, the fibers exhibited a textured and uneven surface, primarily due to the presence of hemicellulose, lignin, and other impurities covering the fibers. These natural surface irregularities contribute to poor surface wet-out and insufficient fiber–matrix bonding. The surface is believed to contain oils, waxes, and dirt. To ensure strong interfacial interactions between the fiber and the polymer matrix, the surface of the fiber must be optimized. Chemical treatment is necessary to remove organic matter and contaminants before incorporating the fibers into the polymer matrix.^96,97^ The cross-sectional analysis shown in Fig. 9b of *R. madagascariensis* fibers reveals that each single fiber is composed of multiple hollow structures of varying shapes. These structures consist of thick microstructures and are enveloped by microfibrils along their longitudinal surface. The covering of microfibrils adds texture and possibly contributes to the durability and adhesion properties of the fibers, which could improve their performance in composite materials or textiles.^98^

**Fig. 10:**
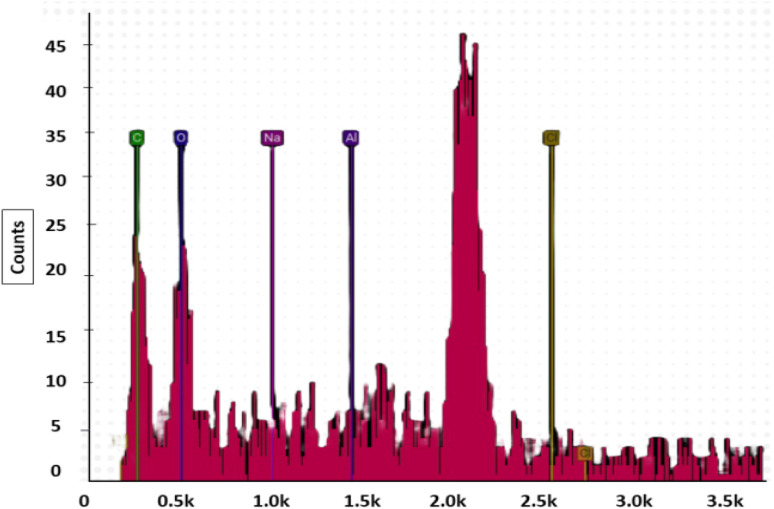
Energy-dispersive X-ray spectroscopy of RMFs.

Fig. 10 shows the elemental quantitative analysis of *R. madagascariensis* fibers, expressed in terms of atomic and mass percentages. In Table 5, quantitative analysis of elements was performed on the basis of atomic percentage and weight. The EDS analysis indicates the absence of nitrogen and sulfur in the fibers.

**Table 5:** EDS analysis of *R. madagascariensis* fibers

Element	Atomic (%)	Weight (%)
Aluminum	9.20	13.58
Carbon	56.73	37.28
Chlorine	16.86	32.71
Oxygen	13.64	11.94
Sodium	3.57	4.4

### 3.8 Chemical composition analysis

**Fig. 11:**
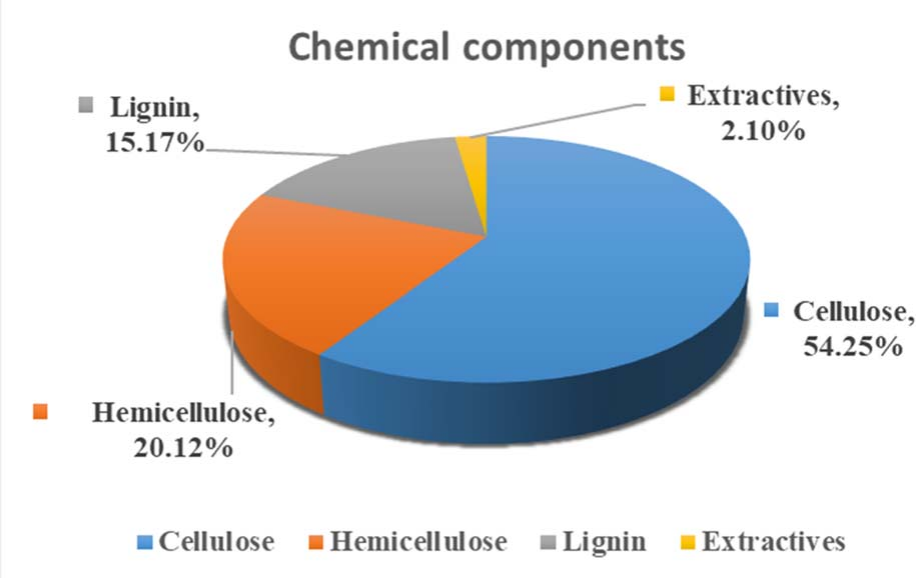
Chemical composition of *R. madagascariensis* fiber.

Identifying the chemical behavior of a substance is essential for understanding its properties, structure, characteristics, and processing capabilities. The composition of a fiber significantly influences these factors. The *R. madagascariensis* fibers were found to contain 15.17% lignin, 20.12% hemicellulose, and 54.25% cellulose. The high cellulose content enhances its suitability for high-value applications and facilitates processing for various purposes. This contributes to improved crystallinity, mechanical properties, biodegradability, hydrolysis resistance, and thermal stability, which are in good agreement with the results shown earlier. Hemicellulose and lignin present in relatively small amounts help regulate the stiffness and bundling of fibers, making them suitable for high-performance composite applications. Additionally, the fiber was found to have 2.10% extractive content. The percentage distributions of these chemical components are illustrated in the pie chart in Fig. 11. A comparison of the chemical composition, mechanical properties, moisture content and thermal stability of the samples is shown in Table 6.

**Table 6:** Comparison of the chemical composition, tensile properties, moisture content and thermal stability of different fibers

Name of fiber	Chemical composition			Tensile strength (MPa)	Moisture content (%)	Thermal stability (°C)	References
	Cellulose (%)	Hemicellulose (%)	Lignin (%)				
RMF	54.25	20.12	15.17	151	9.172 ± 0.601	275	Current work
Coconut tree leaf	27	14	27.7	119.8	4.7	280.1	99
Tamarind	59	22	19	61.16	9.64	264	100
Rosa hybrid bark fiber	52.99	18.49	17.34	352.01	11.60	290	66
Sisal	60–78	10–14.2	8–14	320	20–22	234	101
Pineapple leaf fiber	73.4	7.1	10.5	210–695	9.8	236.6	102, 103
Ficus racemosa	72.36	11.21	10.45	270	6.13	200	104

## 4. Conclusion and outlook

A novel natural fiber was successfully extracted from the leaf stalks of *Ravenala madagascariensis* using environmentally friendly retting methods, including mechanical processing and sun-drying.

X-ray diffraction (XRD) and Fourier-transform infrared spectroscopy (FTIR) confirmed the fiber's structural similarity to other lignocellulosic fibers, reinforcing its classification as a typical plant-derived material.

Chemical composition analysis showed a high cellulose content of 54.25%, making the fiber a strong candidate for applications that benefit from cellulose-rich materials, such as biocomposites and cellulose-based products.

The fineness of the extracted fiber was measured at 33.70 ± 11.72 Tex, which is within the typical range for natural fibers used in various industrial applications.

Scanning Electron Microscopy (SEM) revealed a rough surface with voids and cracks, which may improve fiber-matrix adhesion when used as reinforcement in composite materials.

Thermogravimetric analysis demonstrated that the fiber remains thermally stable up to 275 °C, indicating its suitability for low-to medium-temperature processing conditions.

The fiber exhibited moderate mechanical properties in terms of density, tensile strength, and elongation, supporting its potential use in lightweight composite structures.

The ability to employ RMFs for a variety of purposes, including composite reinforcements and the assessment of their mechanical, chemical, and physical properties, is generally enhanced by this study.

## Author contributions

Mohammad Abul Hasan Shibly – conceptualized, developed the methodology, analyzed the data, and revised the paper. Mohammad Mohsin Ul Hoque – designed the experiments and investigated. Prosenjit Sen – analyzed the data and wrote the draft manuscript. Khandaker Akil, Mahadi Ohi, Md. Maruf Hossain and Md. Masum Mia performed the experiments. Md. Abdus Sabur – analyzed and interpreted the data. Mohammad Junaebur Rashid – experiments, reviewed and edited the manuscript. Mohammad Mahbubur Rahman – contributed reagents and data curation.

## Conflicts of interest

The authors declare that they have no known conflicts of interest or personal relationships that could have appeared to influence the work reported in this paper.

## Data Availability

The data will be made available upon request.
